# Measurement of Low-Concentration Hydrogen in Inert Gas Within a Small Closed Volume

**DOI:** 10.3390/s25123771

**Published:** 2025-06-17

**Authors:** Georgiy A. Ivanov, Dmitry P. Shornikov, Nikolay N. Samotaev, Konstantin Y. Oblov, Maya O. Etrekova, Artur V. Litvinov

**Affiliations:** Moscow Engineering Physics Institute, National Research Nuclear University MEPhI, 115409 Moscow, Russia; ivanov_ga_1016@1511.ru (G.A.I.); dpshornikov@mephi.ru (D.P.S.); kyoblov@mephi.ru (K.Y.O.); moetrekova@mephi.ru (M.O.E.); avlitvinov@mephi.ru (A.V.L.)

**Keywords:** metal hydrides, thermal decomposition, hydrogen, MOSFEC sensor

## Abstract

A technique has been proposed and experimentally tested for measuring the hydrogen concentration in an inert atmosphere within a closed system. This method utilizes a metal-oxide-semiconductor field-effect capacity-type (MOSFEC) sensor under harsh conditions such as exposure to inert gases, pressure fluctuations, and varying temperatures. The measurement is performed during the thermal decomposition of metal hydrides in a liquid sodium environment. The developed measurement technique for determining hydrogen concentration released from metal hydride samples in a system with a closed gas path is cost-effective compared to standardized, resource-intensive open-volume flow measurement methods. The use of the developed MOSFEC sensor technique allows for rapid and efficient investigation of the in situ real-time dynamics of gas release from various metal hydride materials differing in their hydrogen content within a small closed volume. Additionally, this approach enables precise determination of the specific gas release temperatures.

## 1. Introduction

The development of a new generation of heat-stressed technology places increased demands on structural and functional materials located in high-temperature zones. At the same time, a popular technical solution is the use of liquid metals (Na, Li, Pb, etc.). In heat-stressed zones, liquid metal systems have been widely considered for different nuclear energy applications and experimental high-energy setups [1,2,3]. The rather urgent task is to determine the content of various process gases (for example, H_2_) in such a heat-stressed system. Regarding fast neutron reactors, the appearance of H_2_ in the core is due to the presence of fission fragments, (n, α) reactions, for example, on a boron carbide absorber, and the presence of dissolved H_2_ in liquid Na. Exceeding the H_2_ concentration is a negative factor contributing to an increased risk of explosion and material embrittlement [4,5,6].

When studying the “solid body—H_2_—liquid metal” system, several methodological difficulties are noted; for example, determining the precise temperature of H_2_ release and absorption, understanding its release kinetics, and the calculation of the partial pressure of H_2_ within the system [7,8,9]. Obviously, such measurements under the conditions of an operating reactor or heat pipe are extremely labor-intensive, which is associated with a significant volume of coolant and structural materials. At the same time, diffusion tests of individual samples in simultaneous thermal analysis installations or in autoclave tests do not allow for scaling the results or assessing the overall condition of the system. Therefore, this study will be devoted to the selection of instruments and the development of a technique for measuring low concentrations of H_2_ in such confined space conditions.

Measuring small concentrations of H_2_ in closed spaces containing inert process gases (Ar, He, Ne, etc.) is a non-trivial task, often associated with operating conditions that are limiting for the measuring instruments. Therefore, the main factors influencing the selection of sensor element types, considering the limitations of their use, are the change in the sealed volume of the atmosphere of air to inert, which is accompanied by extreme changes in pressure (both vacuum and elevated pressure) and temperature. To measure high concentrations of H_2_ (from 0.1% vol.), the thermal conductometric method is used, since the thermal conductivity of H_2_ differs markedly from the thermal conductivity of other components of the gas flow [10,11,12]. This method is excellent for analyzing two-component mixtures, but for multi-component compositions it is difficult to use this method. To measure small concentrations (up to 0.01% vol.), the simplest solution is the use of various electrochemical cells [13,14,15]. However, this method is very sensitive to temperature, pressure, and the presence of water vapor, since these parameters strongly influence the accelerated aging of the liquid electrochemical cell electrolyte. Considering the above features, to solve many technological problems in measuring H_2_ concentrations, the ability of H_2_ to dissolve and penetrate through metal membranes of Pd and its various alloys is used. In this case, the measurement of H_2_ occurs in a different environment, separated from the total working volume of the chamber, and with a delay of at least the time of H_2_ diffusion through the membrane. Therefore, research aimed at finding means and methods for “in situ” measurement of H_2_ in closed volumes containing inert gases is still relevant. One such method using the MOSFEC sensor manufactured in a ceramic housing for resistance to external factors [16,17] is given below.

## 2. Materials and Methods

**Figure 1:**
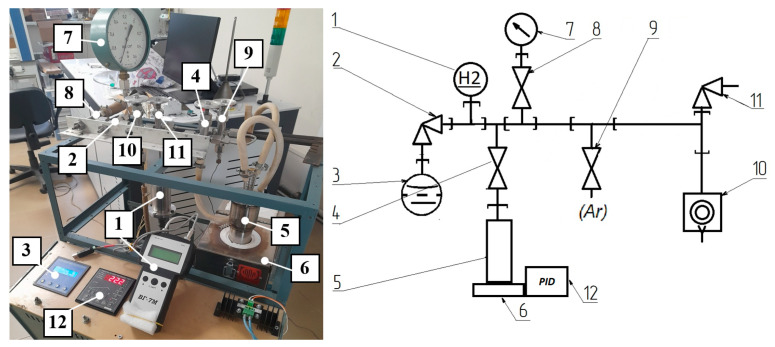
Photo and schematic diagram of the experimental research facility for aging metal hydrides in Na. Hydrogen sensor, 1; vacuum gauge valve, 2; ionization vacuum gauge with hot cathode, 3; valve to the autoclave with Na, 4, autoclave with Na, 5; autoclave heating furnace, 6; wide range mono-vacuum gauge, 7; valve to the backing vacuum pump, 8; Ar input valve, 9; low-vacuum rotary vane pump, 10; gas supply valve to the H_2_ sensor, 11; PID controller, 12.

As part of this study, an experimental research facility has been developed that enables the detection of H_2_ presence above the surface of liquid Na. It comprises an autoclave, a heating system, a H_2_ sensor, gas lines, and vacuum lines. Small quantities (up to 3 g) of TiH_2_ and HfH_2_ were used as sources of H_2_. The schematic representation and photograph of the experimental setup are shown in Figure 1.

The main element of the experimental research facility is an autoclave, into which a sample of TiH_2_ or HfH_2_ was loaded and filled with liquid Na, after which the autoclave was connected to the experimental research facility, then pumping was carried out, and the autoclave was filled with Ar.

To clear the air from the internal volume of the experimental research facility, the air was pumped out until a residual pressure of 3 Pa was reached, and then valve 10 was closed. Valve 9 was opened, and the line was filled with Ar to a pressure of 101 kPa. The pumping and filling stages were repeated 2–3 times to completely remove residual air. This was followed by the final filling of the system with Ar, after which valve 8 was opened, connecting the autoclave with the sample to the main line. After this, heating was conducted at a temperature of 700 °C with a holding period lasting 5 h.

To correctly estimate the amount of H_2_ released from the sample, considering the readings of the MOSFEC sensor, it is necessary to know the exact volume of the system. To determine it, a part of the experimental research facility *V*_1_ of known volume was filled to pressure *P*_1_. The volume was isolated, and the rest of the experimental research facility was pumped down to pressure *P*_2_. Afterwards, the volumes of the unknown and known parts of the experimental research facility were combined, and the resulting pressure *P*_3_ was recorded. The experiment was repeated. As a result, the volume of the entire experimental research facility obtained was equal to *V*_3_ = 270 ± 20 cm^3^ (the ratio of the sample volume to the experimental research facility volume is approximately 1/1000).

**Figure 2:**
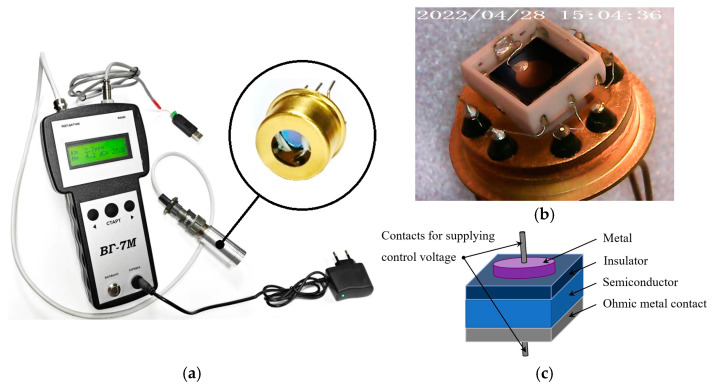
(**a**) The appearance of a gas analyzer based on the MOSFEC sensor. (**b**) MOSFEC sensor assembly on a ceramic base. (**c**) Schematic representation of a capacitor-type MOS structure; for example, a metal Pd electrode with a thickness of 100–200 nm; insulator layer composed of Ta_2_O_5_–SiO_2_, 200–300 nm; semiconductor silicon layer, 4 μm; ohmic platinum contact, 500 nm.

To monitor the H_2_ concentration, a gas analyzer based on the MOS (metal-oxide-semiconductor) capacitor-type sensor was used (Figure 2). The device is suitable for long-term stationary measurements of H_2_ concentration in the air and other gaseous media with a stable background composition. There is a function for displaying current numerical values of H_2_ concentration on the gas analyzer screen and the ability to record, save, and visualize them on a computer in real time using specialized software.

The MOSFEC sensor is designed in a ceramic housing (Figure 2b), manufactured using adaptive laser micro-milling technology [16] specifically for high-temperature applications, aspects of which are discussed in [18]. The basis of the sensor is a capacitive MOS structure (Figure 2c), where the dielectric and metal films of the upper control (H_2_-sensitive) and lower ohmic contacts are obtained using vacuum laser deposition technologies (PLD—pulsed laser deposition). The technology for manufacturing gas-sensitive capacitive MOS structures is described in more detail in [17]. Questions concerning the stability and reproducibility of characteristics and readings of MOSFEC-based hydrogen sensors, including their dependence on operating temperature, were addressed in [19].

The principle of the operation of the gas analyzer is to register the relative change in the electrical capacity of the MOSFEC sensor when H_2_ appears in the analyzed gaseous medium. The dependence of the change in the capacitance of the sensor on the H_2_ concentration ΔC(K_H2_) is a calibration characteristic of the sensor and is determined experimentally. This dependence is nonlinear, which is associated with the kinetics of adsorption of gas molecules on the surface of a solid [20], and the reverse recalculation of K_H2_(ΔC) is performed using the built-in software of the gas analyzer. Two methods are employed for computations:

(1) Piecewise linear approximation with four points intersecting at zero for hydrogen concentration ranges from single-digit to tens of ppm.
(2) Exponential approximation aligned with the initial piecewise linear segment for higher concentration ranges.

The calculation of the concentration value K_H2_(ΔC) is performed depending on the range using the following formulas:

(1) $$K_{H2}=L_{n}\;\times \;∆C,$$

(2) $$K_{H2}=A\;\times \;{exp}^{B\times ∆C},$$

where L*_n_* are coefficients for piecewise linear approximation (*n* = 1…4) and A and B are exponential approximation coefficients that are experimentally determined during instrument calibration. Herein, ΔC = C − C_0_, where C represents the current capacitance sensor reading and C_0_ corresponds to the capacitance at zero hydrogen concentration.

## 3. Calibration of the MOSFEC Sensor

**Figure 3:**
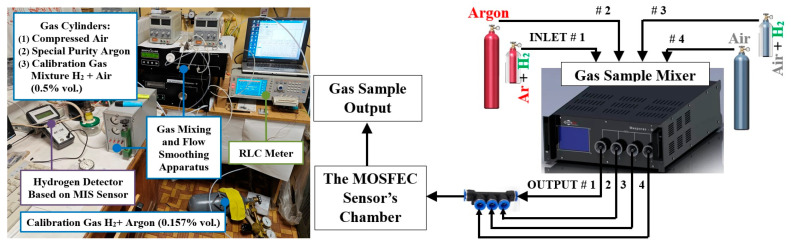
Photo and schematic diagram of the facility for calibrating the MOSFEC sensor’s response to H_2_ in inert atmosphere.

Calibrating the sensor at low concentrations of H_2_ in an inert environment presents a certain difficulty. The appearance of the experimental research facility for calibrating the response of the MOSFEC sensor for H_2_ is shown in Figure 3. The source of reference concentrations was cylinders of calibration gas mixtures: 0.507 ± 0.008% vol. H_2_ in the air and 0.157 ± 0.004% vol. H_2_ in Ar. Cylinders of compressed air and high-purity Ar were used as the test’s zero gas. Mixing of gas flows was carried out at the special gas installation [21] using built-in precision flow controllers and a gas-dynamic mixer [22]. The capacitance-voltage characteristics of the sensor were monitored in order to select the optimal operating point using a precision RCL meter [23]. Summary experimental data on sensor calibration are presented in Table 1.

**Figure 4:**
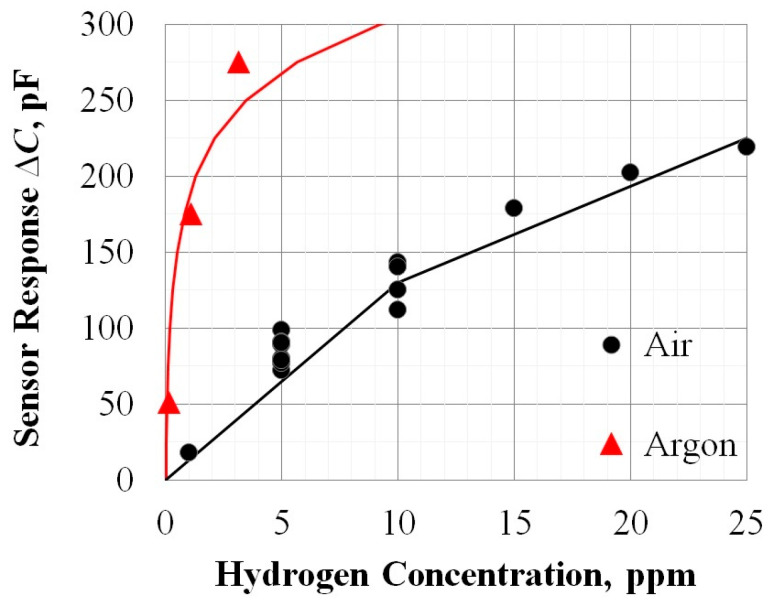
Results of the MOSFEC sensor’s response calibration for a range of low H_2_ concentrations in different gas environments.

As can be seen from the table, provided that the reproducibility of the original sensor signal ΔC is no worse than ±10%, acceptable measurement accuracy is guaranteed only at concentrations up to 100 ppm H_2_. This is due to our experimentally established fact of higher sensitivity to H_2_ in an inert gas compared to air (Figure 4).

A comparison of experimental calibration data for the air and Ar shows that the sensitivity of the MOSFEC sensor to H_2_ in the inert gas environment is significantly higher. So, at a concentration of 5 ppm H_2_, the sensor response in Ar is more than three times higher compared to the air, and at 1000 ppm the increase in sensitivity is no more than 30%. Why does this happen? In [24], by employing density functional theory (DFT) and electron diffraction analysis, the kinetics of hydrogen oxidation reaction (water formation) catalyzed by palladium was investigated. It has been shown that dissociated atoms of H and O compete for open sites on the surface of Pd. Under conditions of equal external concentrations of H_2_ and O_2_, after reaching equilibrium, the surface of Pd ultimately becomes saturated with oxygen atoms due to their higher affinity for adsorption. However, because of their large atomic radius, these adsorbed oxygen atoms could not further diffuse into the lattice of Pd. As a result, they remained on the surface of Pd, and, despite the abundance of hydrogen in the environment, H atoms were unable to effectively enter the lattice of Pd. In our case, these effects can explain why the MOSFEC sensor exhibits greater sensitivity towards hydrogen in an argon medium.

**Figure 5:**
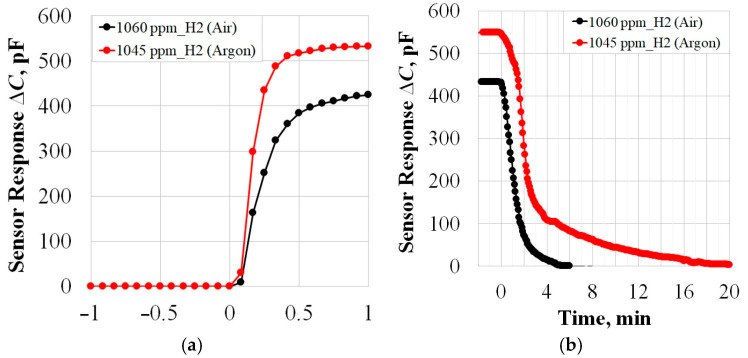
(**a**) The MOSFEC sensor’s response rate to the supply (**a**) and removal (**b**) of H_2_.

The response rate to the supply of H_2_ is *τ*_0.9_ ≈ 30 s (Figure 5a) and does not depend on zero gas, and the relaxation rate (return to zero) in Ar is significantly lower: *τ*_0.1*_Ar*_ ≈ 9 min; *τ*_0.1*_Air*_ ≈ 3 min (Figure 5b). This is because, as mentioned above, water vapor formation on the catalyst’s surface is one possible mechanism for removing hydrogen from palladium [24]. Typically, this process involves numerous parallel and sequential reactions resulting either in H_2_O or H_2_O_2_ [25], with H_2_O formation being thermodynamically more favorable [26]. Due to the absence of oxygen in the argon atmosphere, the contribution of this mechanism to the relaxation process is eliminated, thus slowing down the sensor response.

**Figure 6:**
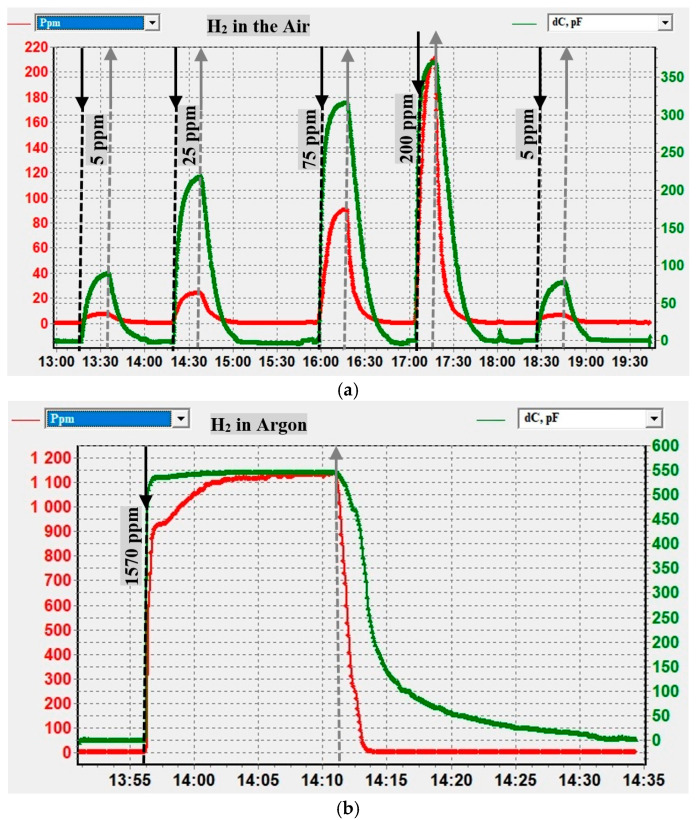
Verification of the MOSFEC sensor’s calibration results for H_2_: (**a**) 5, 25, 75, 200, and 5 ppm in the air; (**b**) 1570 ppm in Ar. Notations: red curve (left *y*-axis)—measured instrument values of H_2_ concentration [ppm]; green curve (right *y*-axis)—experimental data of relative change in sensor capacitance [pF]; *x*-axis—time scale in 24 h format hour: minute.

Verification of the MOSFEC sensor’s graduation results was carried out using calibration gas mixtures in two stages with the corresponding coefficients inserted into the gas analyzer memory: first in air and then in Ar. The results of the first stage with the supply of reference H_2_ concentrations of 5, 25, 75, 200, and again 5 ppm in the air are shown in Figure 6a: green curve—response of the MOSFEC sensor ∆*C*, pF; red curve—H_2_ concentration readings, ppm, calculated using the gas analyzer software. The uncertainty of reference H_2_ concentrations did not exceed ± 2% and was determined primarily by the error of the cylinder containing the certified gas mixture and the error in setting and measuring flow rates in the special gas installation’s channels for the dynamic mixing of flows. The error in measuring concentration by the MOSFEC sensor was no more than 20%.

The results of the second stage of testing in an Ar environment are shown in Figure 6b. As can be seen, the values of the initial useful signal of the MOSFEC sensor—changes in capacitance ΔC—correspond to good reproducibility of the readings (see Table 1). However, due to the previously indicated features of the reverse recalculation of K_H2_(ΔC) and the nonlinearity of the calibration dependence, the recorded readings of large H_2_ concentrations are 27% lower than the reference value, which is beyond the permissible measurement error. For the same reason, the dynamics of the reaction to H_2_ are also significantly different if assessed by the increase in concentration readings (*τ*_0.9_*_*_KH2_ > 3 min, Figure 6b, red line) or the capacitance change signal (*τ*_0.9*_*ΔC_ ≈ 30 s, Figure 6b, green line, also Figure 5a). Thus, due to the weak differentiation of sensor responses to high concentrations of H_2_ in an Ar environment, the recommended reliable range for measuring H_2_ in this study was no more than 300 ppm. Nevertheless, for cases where the recommended concentration range is exceeded, it will remain possible to qualitatively assess gas evolution over time, which is also useful at the current stage of research for the relative comparison of data for different samples of metal hydrides.

After calibrating the sensor for hydrogen detection, we examined the effect of increasing pressure up to 8 atmospheres on two types of zero gases: air and argon. It should be noted that due to technological limitations in producing pressurized gas mixtures in cylinders, even with zero-gases, the H_2_ concentration unfortunately does not equal zero. According to the specifications provided with the cylinders used in this study, the air zero-gas contained no more than 2 ppm of hydrogen as an impurity, while the argon zero-gas had a residual hydrogen content of 0.8 ppm. Consequently, under the influence of 8 atmospheres of pressure, the sensor’s capacitance increased by approximately 40 pF when exposed to air, which, according to our calibration data (Figure 4), appears like the response from background concentrations of residual hydrogen present in the cylinder. The reaction of the sensor to excess pressure of 8 atm in argon was about 3.5 times higher. This also resembles the response to residual H_2_ in the zero-gas cylinder, considering the observed enhancement of sensitivity in an inert gas environment. Therefore, it can be concluded that pressure itself has no direct impact on the measurements made by the MOSFEC sensor.

## 4. Results

To qualitatively and quantitatively evaluate the processes of H_2_ evolution from metal hydrides upon heating up to 700 °C, as well as to investigate potential hydrogen uptake by sodium, we conducted a series of experiments whose results are presented below.

**Figure 7:**
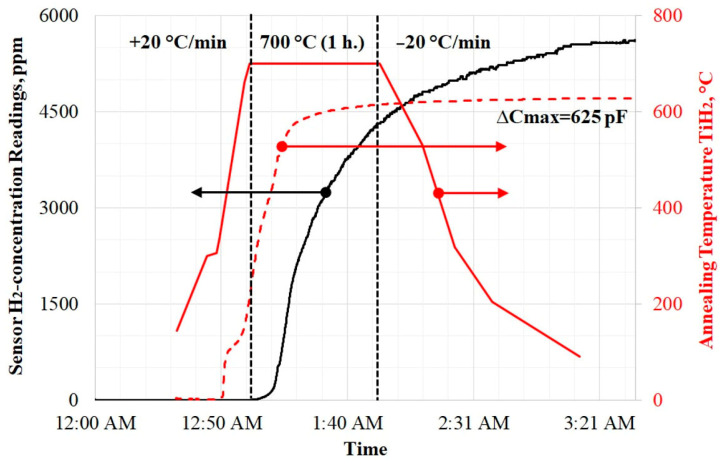
Dynamics of H_2_ release from a TiH_2_ sample in the case of heating and holding at 700 °C.

Figure 7 shows data illustrating the yield of H_2_ from a TiH_2_ sample. The red dashed line corresponds to the primary signal ΔC of the MOSFEC sensor (see the coordinate axis on the right, which is aligned with the temperature heating sample value axis). This ΔC data is used to calculate and plot the graph of H_2_ concentration (the coordinate axis on the left). Note that in this experiment, the sensor capacitance readings were ∆*C* > 600 pF, which means that the recorded concentration values are approximate. Despite this, experience shows that with this heating mode, the sensor readings reach saturation and the amount of H_2_ in the experimental research facility volume stabilizes within 2–3 h. It is evident that the increase in ΔC (and hence the appearance of H_2_) begins when the heating temperature reaches ≈300 °C. However, the significant rise in H_2_ concentration occurs only upon reaching 695 ± 5 °C, which aligns with the literature data [27,28]. At this point, ΔC already exceeds 200 pF, and, according to the calibration in Figure 4, this corresponds to merely ≈1 ppm of H_2_.

**Figure 8:**
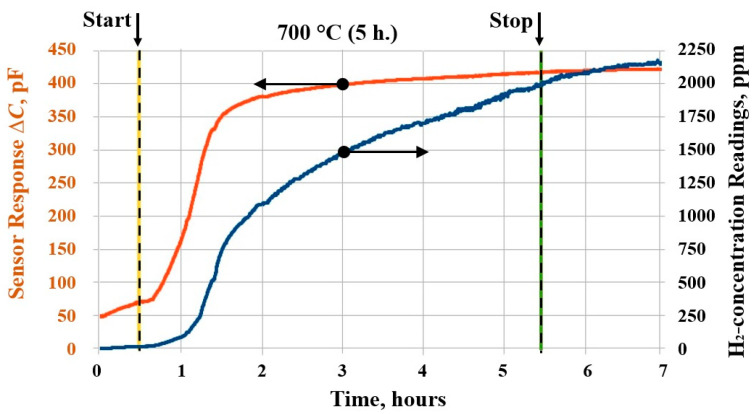
Graph of changes in H_2_ concentration when HfH_2_ is kept in Na at 700 °C for 5 h.

In Figure 8, the experimental dependence for HfH_2_ sample is shown. Based on the readings of the MOSFEC sensor, the beginning of H_2_ evolution for HfH_2_ occurs at a temperature of 645 ± 5 °C, which generally aligns with the data in the phase diagram.

Thus, the high sensitivity of the MOSFEC sensor in argon would likely enable detection of H_2_ concentrations at the level of parts per billion (ppb). However, the practical challenge here lies in the lack of calibration methods for such ultra-low H_2_ concentrations.

## 5. Discussion

Analysis of the results obtained, including stabilization of the maximum readings of H_2_ concentration after 5 h of the experiment, suggests that not all H_2_ is released from the hydride samples. Subsequent weighing revealed a mass loss of the samples ranging from 1 to 3 mg (not exceeding 0.1 wt.%), confirming this hypothesis. This phenomenon appears to be attributed to the establishment of thermodynamic equilibrium within the metal hydride–H_2_ system, which is linked to an elevation in the partial pressure of H_2_ above liquid Na. During the experiments, the procedure for converting the MOSFEC sensor’s readings was refined and adjusted, considering the characteristics of the capacitance difference signal and ensuring proper calibration of the zero point. When working in argon, we encountered poor differentiation of sensor readings for H_2_ concentrations above 100 ppm. It is likely that in an inert gas environment, the MOSFET sensor will be suitable for measuring H_2_ concentrations no higher than tenths of a percent volume fraction (% vol.). However, for solving the task described in this article, it is more important to detect the moment when hydrogen begins to escape from samples, which requires high sensitivity. If there is still a need to expand the operating concentration range, the authors would suggest using other types of detectors together with MOSFET, including those based on thermal conductivity.

It was shown that the presence of liquid Na and its vapor does not in any way affect the performance of the sensor, which is important for longer tests in the future at different heating temperatures of hydride samples. The study also shows that, despite the relatively simple nature of the measuring equipment, experiments on the thermal decomposition of metal hydrides within a closed gas circuit using the MOSFEC sensor represent an acceptable alternative to standardized experiments performed in an open system equipped with high-precision instruments like a simultaneous thermal analyzer coupled with a quadrupole mass-spectrometer. The developed experimental setup has confirmed its performance and is ready for further tests; for example, to study the extremely low content of H_2_ dissolved in various metals.

## 6. Conclusions

A technique has been proposed and experimentally tested for measuring the H_2_ concentration in an inert atmosphere within a closed system under harsh conditions (inert gases; pressure and temperature dropping) during the thermal decomposition of metal hydrides using a MOSFEC sensor. The research facility has been designed that enables experiments to be carried out and hydride samples to be heated up to 700 °C in the presence of liquid Na; that is, under conditions close to real nuclear reactor operating conditions. This sets the setup apart from standardized resource-intensive open-volume flow methods. The developed MOSFEC sensor technique will enable rapid and efficient investigation of the real-time gas-release dynamics from metal hydride samples differing in hydrogen content within a closed small volume, allowing precise determination of the gas-release temperatures. The described setup and the MOSFEC sensor used may have more applications than those presented in the paper—there are various built-in online monitoring systems for complex technical objects with inaccessible or very dangerous parts characterized by high temperatures, pressure drops, and dangerous chemical reactions with oxygen during depressurization.

## Figures and Tables

**Table 1:** The calibrating data of the MOSFET sensor’s response to H_2_ in an Ar environment.

K_H2_, ppm	0.2	1.1	3.1	65	350	640	1045	1570
**ΔC, pF**	50 ± 5	175 ± 20	275 ± 30	400 ± 40	530 ± 50	535 ± 50	545 ± 40	550 ± 50

## Data Availability

The original contributions presented in this study are included in the article. Further inquiries can be directed to the corresponding author.
